# The Versatility of the *Helicobacter pylori* Vacuolating Cytotoxin VacA in Signal Transduction and Molecular Crosstalk

**DOI:** 10.3390/toxins2010069

**Published:** 2010-01-15

**Authors:** Steffen Backert, Nicole Tegtmeyer

**Affiliations:** Ardmore House, School of Biomolecular and Biomedical Sciences, Belfield Campus, University College Dublin, Dublin-4, Ireland; Email: nicole.tegtmeyer@ucd.ie (N.T.)

**Keywords:** *Helicobacter pylori*, signalling, type IV secretion, CagA, pathogenicity island, vacuolating cytotoxin, VacA, lipid rafts

## Abstract

By modulating important properties of eukaryotic cells, many bacterial protein toxins highjack host signalling pathways to create a suitable niche for the pathogen to colonize and persist. *Helicobacter pylori* VacA is paradigm of pore-forming toxins which contributes to the pathogenesis of peptic ulceration. Several cellular receptors have been described for VacA, which exert different effects on epithelial and immune cells. The crystal structure of VacA p55 subunit might be important for elucidating details of receptor interaction and pore formation. Here we discuss the multiple signalling activities of this important toxin and the molecular crosstalk between VacA and other virulence factors.

## 1. Introduction

*Helicobacter pylori* (*Hp*) is a highly successful pathogen that is estimated to persistently colonize the stomach of about half of the human world population. This bacterium has been recognized as the causative agent of chronic gastric inflammation, peptic ulcer disease, mucosa-associated lymphoid tissue (MALT) lymphoma or gastric cancer [1,2,3,4,5,6]. The clinical outcome of *Hp* infections is determined by a complex scenario of host-pathogen interactions. The cellular and molecular mechanisms used by *Hp* to subvert host defences have been investigated intensively over the last few years. Disease development is dependent on several parameters including both the genotype of the bacteria and genetic predisposition of the host, as well as environmental factors [7,8,9,10,11,12]. *Hp* isolates from different geographical origin are surprisingly diverse both in their genome sequences and their pathogenicity. Dozens of bacterial factors have been shown to influence *Hp* pathogenesis including the flagella, urease, catalase, neutrophil-activating protein NapA, peptidoglycan, several adhesins (e.g., SabA, BabA, AlpA/B, OipA) and many others [1,13,14,15,16]. They can be classified as colonization factors (also called pathogenicity-associated factors) and virulence factors having direct cell-damaging capabilities. However, the two major disease-associated components are the vacuolating cytotoxin (VacA) and the *cag* (cytotoxin-associated genes) pathogenicity island (*cag*PAI). The gene encoding *vacA* is present in virtually all *Hp* strains, which suggests that the production of VacA plays an important role in the colonization or persistence of *Hp* in the human stomach [1,7,8,9,17]. The *cag*PAI is a 40 kb stretch of DNA, which was acquired by a horizontal DNA transfer event from yet unknown donor, and encodes components of a sophisticated type IV secretion system (T4SS). This T4SS forms a pilus for the injection of virulence factors into host target cells such as the CagA effector protein [7,11,12,18,19,20]. Injected CagA mimics a host cell protein in binding and activation of multiple signalling factors and induces actin-cytoskeletal rearrangements (cell elongation), disruption of cell-to-cell junctions (cell scattering) as well as proliferative, pro-inflammatory and anti-apoptotic nuclear responses [11,20,21,22,23]. In addition, *Hp* induces several T4SS-dependent but CagA-independent signalling events such as the activation of epidermal growth factor receptor EGFR [24,25] and the small Rho GTPase Rac1 [26,27,28]. The role of CagA and the T4SS have been discussed in numerous recent reviews [7,11,12,20]. Here we review the recent findings on the VacA structure and the various pathways how VacA highjacks host cell signalling cascades. We will also discuss a set of novel findings on the molecular crosstalk between VacA and CagA.

## 2. VacA Expression, Secretion, Maturation and Allelic Variation

**Figure 1:**
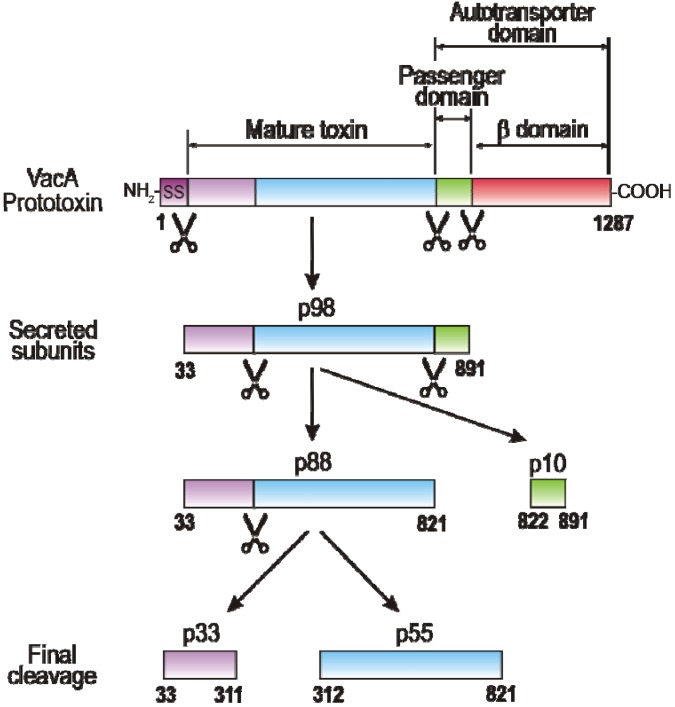
VacA domain structure and processing products. The amino-terminal signal sequence (SS) and carboxy-terminal domain are cleaved from the full-length 135-140 kDa VacA protein to yield a 98 kDa secreted toxin (p98) that is transported into the extracellular space *via* the autotransporter mechanism. Proteins of this autotransporter family are able to mediate their own secretion without the need of additional bacterial proteins. The 33 kDa carboxy-terminal beta-barrel domain of VacA is predicted to insert into the outer membrane and to form a channel, through which the mature VacA toxin is secreted. The secreted toxin can be processed into the mature toxin (p88) and p10. The mature, secreted p88 subunit can undergo further limited proteolytic cleavage to yield two additional fragments, p33 and p55 [7,8,32]. The latter two processing products are considered to represent the functional domains or subunits of VacA.

An early work by Leunk and colleagues [29] discovered that broth-culture supernatants of *Hp* strains contained a proteinaceous cytotoxin that induced cytoplasmic vacuolation when incubated with eukaryotic cells *in vitro*. Multiple cell types from human and animal species were susceptible to its toxic activity, albeit with great differences. The structural organization of VacA closely resembles that of IgA protease-like factors secreted by pathogenic *Neisseriae* and *Haemophilus* species [9], which are today known as the autotransporter or type V secretion family of proteins [18]. Like many other classical autotransporters, VacA contains a 33-amino acid signal sequence and undergoes cleavage during the transport across bacterial membranes (Figure 1). *VacA* genes are predicted to encode a translated protein of about 135-145 kDa depending on the strain. However, VacA proteins of this molecular weight have never been reported [8]. Instead, three VacA protein spots of the *Hp* strain 26695 were identified by two-dimensional gel electrophoresis (2-DE). The most predominant VacA protein spots on 2-DE gels were approximately 95.7 kDa and 87.9 kDa in size, and were found both in fractions of the *Hp* membrane and culture supernatant [30,31]. In addition, a 10.5 kDa VacA spot was found in the secreted protein fraction [30]. Mass spectrometry of the identified peptides are consistent with a model that the 95.7 kDa VacA protein is the secreted toxin which is then cleaved into the 87.9 kDa mature protein (p88) and the 10.5 kDa passenger domain (p10, also known as the alpha-protein) (Figure 1). The exact cleavage site between p88 and p10 was detected between position 991 and 992 [30]. The processed beta-domain of the autotransporter subunit was not detected by 2-DE, but has been observed in total cell lysates [32]. However, the mature translocated p88 domain can undergo further proteolytic cleavage to yield two fragments, known as p33 and p55 (Figure 1, bottom). The latter two processing products are believed to represent the functional domains of VacA (see below). Thus, VacA may exhibit some properties resembling those of classical A/B-toxins. Interestingly, while cleavage between p33 and p55 subunits has been seen *in vitro* [33], no cleavage *in vivo* could be detected [34]. A possible explanation is that the rate of VacA cleavage *in vivo* is too low to be observed. In some studies, VacA and its domains have slightly different sizes, but we will use the p33 and p55 nomenclature throughout this review. However, the basis of *vacA* size and sequence variation was found in several alleles that have been identified in the signal region (genotypes s1 and s2) and in the mid region (genotypes m1 and m2), occurring in all possible combinations [1,7,8,9]. In addition, two intermediate region variants (called i1 and i2) were identified as other important determinants of VacA toxicity [35]. VacA proteins of the s2 type are inactive in assays for the vacuolating phenotype, while type s1/ml alleles produce extensive vacuolation in a large array of cell categories and type s1/m2 allels induce detectable vacuolation in a more limited range of cell types, which is in concurrence with its location in p55 as the cellular binding domain of VacA [36,37].

## 3. Domains of VacA and Crystal Structure of the p55 Subunit

**Figure 2:**
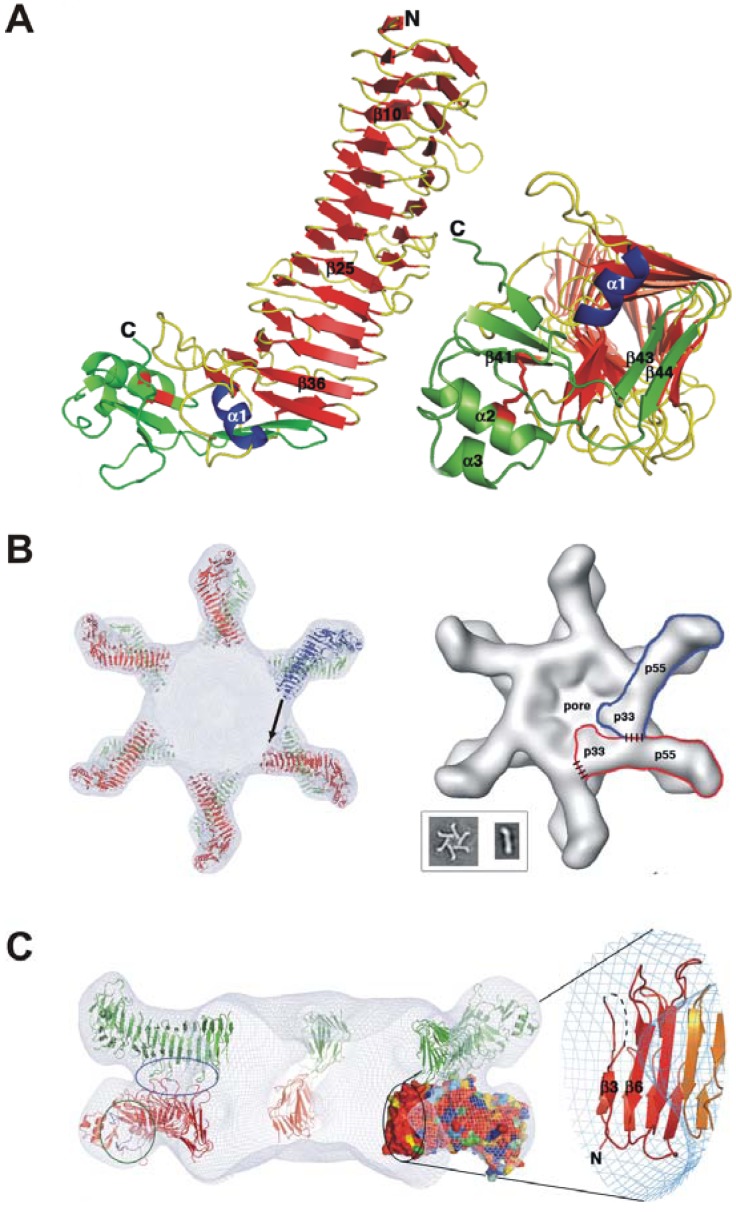
Molecular structure of VacA. (A) The p55 domain of VacA adopts a β-helix structure that is composed of three parallel β-sheets connected by loops of varying length and structure [51]. The carboxy-terminal domain contains a mixture of α/β secondary structure elements and contains a disulfide bond. The carboxy-terminus of the β-helix (right, rotated by 90°) is capped by a β-hairpin from the carboxy-terminal domain and the α1−helix located in one of the long helix loops. (B) Docking the p55 crystal structure into a 19-Å cryo electron microscopy map of the VacA dodecamer results in a model for oligomerization. Twelve p55 subunits are shown docked into a 19-Å cryo-EM map of a VacA dodecamer [44]. An arrow is shown to indicate the space that the upper molecule will occupy if p33 extends the β-helix structure of p55. The inset shows EM images of a VacA hexamer and a VacA monomer [44]. The shape of a VacA hexamer (*inset*) is similar to the shape of a single layer within the dodecamer [44]. The rod-like shape of the p88 monomer (*inset*) supports a model in which the β-helix of p55 will extend into p33. (C) The view from the left structure in panel B is rotated by 90°. This figure was kindly provided by Dr. Borden Lacy and originally published by Gangwer *et al*. [51] with kind permission of the National Academy of Sciences, USA.

In response to acidic pH, VacA undergoes a structural change, which increases its cellular activity and renders it resistant to proteolysis by pepsin [38]. The relevance of the pH, however, is still not fully understood because VacA has also been shown to be activated at alkaline pH [39]. In fact, it appears that only highly purified VacA is poorly active and needs to be activated by pH change, but this is not necessary during infection or when VacA is simply collected from the bacterial supernatant (broth culture filtrate). Thus, VacA secreted by the bacteria is most likely fully active independently of the luminal gastric pH. This is in agreement with the fact that the mucus layer close to the gastric epithelium, in which *Hp* resides, is at a mild acidic pH. However, the secreted p88 subunit of VacA can assemble into water-soluble oligomeric structures [33,40], and can insert into planar lipid bilayers to form anion-selective membrane channels [41,42,43]. These structures resemble a flower-like configuration (Figure 2B). Interestingly, low pH treatment of these oligomeric structures break them apart releasing VacA monomers [40]. The first reported high-resolution images were 19-Å cryo electron microscopy maps of VacA dodecamers [44], and atomic-force microscopy and electrophysiological studies implied that VacA membrane channels are single-layered structures [41,43]. When the p33 and p55 domains are expressed separately and then mixed, p33 and p55 can physically interact and restore vacuolating activity in epithelial cells [45,46,47]. The p33 subunit contains a hydrophobic stretch (amino acids 6-27) which is involved in pore formation and/or membrane insertion [48,49], whereas p55 contains one or more cell interaction sites [36,37,50]. Recently, the 2.4-Å crystal structure of p55 has been published [51]. The structure is predominantly a right-handed parallel β-helix, a feature which is characteristic of autotransporter passenger subunits but unique among other known toxins (Figure 2A-C). Notable features of p55 include disruption of β-sheet contacts which result in five β-helix subdomains and a carboxy-terminal domain that contains a disulfide-bond. Gangwer *et al.* [51] proposed an oligomerization model in which p33 interacts with the amino-terminal portion of p55 from the neighboring subunit. Regions of contact between p33 and p55 are depicted with dashed lines (Figure 2B, right structure). On the left in Figure 2C, the upper circle highlights three loops that mediate p55-p55 contacts between the two layers. The lower circle indicates the two loops that line the conserved pocket that were proposed as a common receptor-binding site [51]. This pocket is located on the side of the molecule and would therefore be accessible in both single-layered and bilayered forms of the toxin. The circle in the middle contains the other conserved surface in p55. This surface protrudes into the central ring of the VacA oligomer and would be accessible to the molecule for contacts that could mediate oligomerization within a hexameric or heptameric plane. The enlarged section to the right in Figure 2C shows the secondary structures that contribute to this surface. Again, p55 contains the aforementioned m1/m2 alleles, and investigation of VacA sequences from unrelated *Hp* strains allowed the identification of structural surface features which may be important for interactions with host cell receptors [51].

## 4. VacA Targets Various Surface Factors Both on Epithelial and Immune Cells

**Figure 3:**
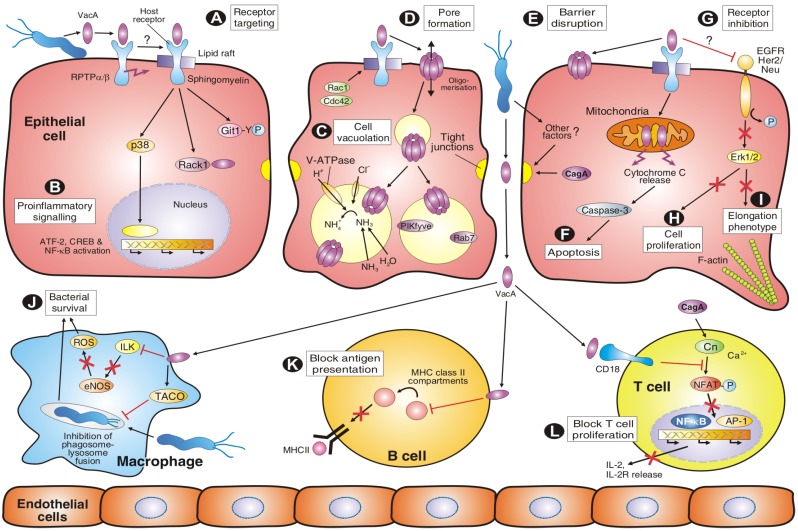
Model of the various activities of VacA to induce altered host cell signalling in epithelial and immune cells which contributes to persitent *Hp* colonization in the human stomach.

The cellular effects of VacA have been investigated intensively both *in vivo* and *in vitro*. A simplified model for the complex interactions of VacA with multiple cell types and downstream signalling cascades is presented in Figure 3A-L, while known host cell binding partners are summarized in Table 1. Like many other bacterial toxins, VacA interacts with the plasma membrane of susceptible cells as a first step during intoxication (Figure 3A). Following cell binding, VacA is internalized by a pinocytic-like and clathrin-independent mechanism [52,53]. A remarkable feature of VacA is that it can target multiple cell-surface components *in vitro* or on epithelial cells including RPTP-alpha [54], RPTP-beta [39,55], various lipids [56,57,41], heparin sulphate [58], sphingomyelin [59], fibronectin [60], the epidermal growth factor receptor (EGFR) [61] as well as integrin β2 (CD18) on T cells [62]. Determining which of these factors is required for VacA uptake and subversion of cellular signalling has been a challenging topic of research and is still not fully studied for each factor. However, the importance of RPTP’s for VacA-dependent activities has been investigated in much detail. For example, VacA causes gastric injury in wild-type mice but not in the RPTP-beta knockout mice [55]. This indicates that interaction of VacA with RPTP-beta on the surface of gastric epithelial cells is mechanistically important in VacA-induced gastric injury. Remarkably, VacA can still induce vacuolation in cultured gastric epithelial cells from RPTP-beta knockout mice or in G401 human kidney cells, which do not express RPTP-beta [54,55], but inhibition of RPTP-beta expression by silencing oligonucleotides in HL-60 cells downregulated VacA-induced vacuolation [63]. These data suggest that RPTP-beta exhibits crucial functions in the process of VacA-induced signalling but not necessarily vacuolation itself. Addition of VacA to target cells has been also shown to induce the tyrosine phosphorylation of G-protein-coupled receptor-kinase-interacting protein-1 (Git-1) and lipid-raft dependent downstream signalling [55,64]. In line with these observations, VacA was reported to bind to the QTTQP-motif in the extracellular domain of RPTP-beta, which was also necessary for inducing Git-1 phosphorylation [54]. Another reported consequence of VacA-binding to RPTP-beta is detachment of primary murine gastric epithelial cells from the extracellular matrix [55]. Furthermore, VacA has been shown to interact with lipid preparations and artificial membranes, but the specificity and importance of VacA-lipid interactions for toxin function was unknown until recently. A new study provided evidence that sphingomyelin modulates the sensitivity of epithelial cells to VacA [59]. Sphingomyelin is a key structural component of the cell membrane (in particular lipid rafts), while at the same time serving a functional role, as it is the parental compound of various lipid mediators. In fact, sphingomyelin was shown to be required for VacA’s association with lipid rafts on the plasma membrane and binding to cells, suggesting that sphingomyelin functions as a receptor molecule of VacA on the epithelial cell surface [59]. However, VacA does not only interact with gastric epithelial cells, but also with immune cells including B cells, T cells and macrophages (Figure 3J-L). Interestingly, VacA efficiently enters activated, migrating primary human T lymphocytes by binding to the integrin β2 (CD18) receptor and exploiting the recycling of LFA-1, the lymphocyte function-associated molecule-1 [62]. LFA-1-deficient Jurkat T-cells were resistant to vacuolation, and genetic complementation of LFA-1 restored the sensitivity of cells to VacA. Remarkably, VacA targeted human CD18 for cell entry, but not its murine counterpart. These findings are consistent with the species-specific adaptation of *Hp* in its human host [62].

**Table 1:** Host binding partners of VacA and proposed function ^a^.

VacA binding partners	Proposed role in VacA-host interactions	Methods used	Experimental evidence^b^	Reference
EGF receptor	Receptor on epithelial cells	Antibody blocking, IP	Infection *in vitro*	[61]
Fibronectin	Binding of *H. pylori* to host cells	Binding and cell adhesion and assays	Binding *in vitro*	[60]
Glycosphingolipids	Binding partner for VacA internalization	Binding assays, Chromatography, MS	Binding *in vitro*	[65]
Heparin sulphate	Receptor/co-receptor on epithelial cells	SPR-based biosensor studies	Binding *in vitro*	[58]
Integrin β2 (CD18)	Receptor on T cells	Flow cytometry, IF, live cell imaging, use of knockout cells	Treatment of cells *in vitro,* Infection *in vitro*	[62]
Lipid bilayers	Low pH-triggered pore formation	AFM	Binding *in vitro*	[41]
Lipid rafts	Putative docking and entering site at cell surface	Cell fractionation, flow cytometry, IF, MCD inhibitor studies	Treatment of cells *in vitro*	[66,67,64]
Lipid vesicles	Binding and host cell entry of the toxin	Light scattering, energy transfer studies	Binding *in vitro*	[56]
Phospho-lipids	Binding and host cell entry of the toxin	ANS-binding studies, Photolabelling	Binding *in vitro*	[57]
RPTP-alpha	Receptor on epithelial cells	IP, MS, antisense silencing studies	Infection *in vitro*, Treatment of cells *in vitro*	[54,68]
RPTP-beta	Receptor on epithelial cells, receptor for Git1 phosphorylation	Flow cytometry, IF, IP, siRNA, use of knockout mice and cells	Binding *in vitro*, Infection of mice and cells	[39,63,55]
RACK-1	Yet unknown	PD, Y2H	Binding *in vitro*	[69]
Sphingo-myelin	Receptor on epithelial cells	Binding studies, ELISA, flow cytometry, treatment with sphingomyelinase	Binding and treatment of cells *in vitro*	[59]

^a^ Abbreviations used: ANS (1-Anilino-8-naphtalensulfonate), AFM (atomic force microscopy), EGF (epidermal growth factor), ELISA (enzyme-linked immunosorbent assay), IF (co-localization by immunofluorescence), IP (immunoprecipitation), MCD (methyl-β-cyclodextrin), MS (mass spectrometry), PD (pull-down), siRNA (silencing RNA knockdown), SPR (surface plasmon resonance), Y2H (yeast two-hybrid). For other abbreviations, see text.

^b^ Term explanation: Binding *in vitro* (purified and/or recombinant VacA was incubated with the factor of interest *in vitro*); Infection *in vitro* (*Hp* infection of cultured host target cells); Infection of mice (infection of wild-type and knockout mice); Treatment of cells *in vitro* (purified or recombinant VacA was applied externally to cultured host cells *in vitro*, and then binding of the factor of interest was investigated).

## 5. VacA Activities on Endosomal Maturation and Cell Vacuolation

As mentioned above, the first identified activity of VacA was its ability to induce vacuolation in numerous eukaryotic cell types [29]. A current model for vacuole formation suggests that VacA binds to the plasma membrane of host cells, forms anion-selective channels in the plasma membrane, becomes internalized into endosomes and the vacuoles finally arise by swelling due to osmotic imbalance of the late endosomal compartments (Figure 3C). In fact, VacA-induced vacuoles constitute hybrid compartments of late endosomal origin that contain some lysosomal markers, and their formation strictly depends on the presence of weak bases such as ammonia, which can be provided by urease activity [34,70]. To achieve this goal, VacA is first pinocytosed in a manner dependent on the small Rho GTPase Cdc42 [53], and is subsequently routed into early endosomal compartments which are enriched with glycosylphosphatidylinositol (GPI)-anchored proteins [52,53]. These early endosomes are also associated with dynamic polymerised (filamentous) actin structures [71]. The CD2-associated protein (CD2AP), a docking protein implicated in intracellular trafficking, bridged the filamentous actin with early endosomes containing VacA. Interestingly, CD2AP regulated these actin structures and was required to transfer VacA from early to late endosomes and vacuolation [71]. Vacuole formation further depends on the presence and activity of a number of other signalling factors including V-ATPase [72], Rab7 [73] and Rac1 [74]. Recent studies showed that at least the role of Rac1 in vacuole formation is due to the fact that Rac1 is required for binding of VacA to the cell membrane [53]. However, protein kinase PIKfyve is also necessary for vacuole production, and microinjection of its substrate phosphatidylinositol-3,5-bisphosphate (PIP2) was demonstrated to be sufficient for vacuole formation [75], whereas involvement of the SNARE protein syntaxin-7 is controversial [76,77]. Also, the role of dynamin in large VacA-dependent vacuole formation is controversial. Dynamin has been shown to play either no role [52] or is fully necessary for cell vacuolation [78]. When expressed intracellularly, the minimum segment of VacA required for vacuolating activity comprises the entire p33 subunit and about 110 amino acids from the amino-terminus of p55 [45,79]. Vacuolating activity also requires di- or oligomerization of VacA [80,81] and depends on the hydrophobic amino-terminus (about 30 amino acids) containing three tandem GXXXG-sequences which are transmembrane dimerization motifs [49,82]. These requirements suggest that channel formation is a prerequisite for vacuolation. This conclusion is also supported by the observation that small anion-channel inhibitors effectively block vacuole production [83].

## 6. Amino-Terminal VacA Targets Mitochondria to Induce Apoptosis

Infection of epithelial target cells with *Hp* has been associated with both increased and reduced levels of apoptosis [1,2,7]. *In vitro*, *Hp* reproducibly stimulates apoptosis in infected gastric epithelial cells, and this has also been seen in tissues isolated from infected patients or animal models [1,2]. Early studies indicated that purified VacA induced mitochondrial damage as indicated by a significant drop in ATP levels within cells [84], and was then discovered to induce apoptosis by a mitochondrial pathway [85]. In the latter study, HEp-2 cells were transfected with different VacA constructs, and the p33 subunit was found to localize specifically to mitochondria, whereas p55 remained cytosolic. When purified VacA was co-incubated with isolated mitochondria, p33 (but not p55) was translocated into these organelles. In addition, transient expression of p33-GFP or full-length VacA-GFP in HeLa cells induced the dramatic release of cytochrome *c* from mitochondria and activated apoptotic caspase-3 (Figure 3F), as determined by the cleavage of poly-ADP-ribose polymerase, PARP. Cleavage of PARP was antagonized specifically by co-transfection of DNA vectors expressing Bcl-2, a factor known to inhibit mitochondria-associated apoptotic signals [85]. These observations were further supported by infection studies using wild-type *Hp* and Δ*vacA* mutant strains [86,87]. Remarkably, the s1/m1 allel of VacA induced high levels of apoptosis while s2/m1 toxin and truncated VacA mutants lacking the hydrophobic region near the amino-terminus did not. These results suggest that VacA can trigger gastric epithelial cell apoptosis and implicate that differences in levels of gastric mucosal epithelial apoptosis among *Hp*-infected individuals may arise from strain-dependent *vacA* sequence variation. Interestingly, studies in MKN-28 cells showed that, unlike cell vacuolation, neither apoptosis nor change of mitochondrial transmembrane potential induced by VacA require ammonia [88]. The latter findings make unlikely the hypothesis that ammonia-dependent swelling and rupture of endosomal vesicles in which VacA is sequestered after cellular uptake may allow the toxin to reach mitochondria and trigger apoptosis, and therefore requires a different pathway.

## 7. VacA and Other Factors Affecting the Transepithelial Resistance of Polarized Cell Monolayers

An important feature of intact polarized cells is the development of transepithelial electrical resistance (TER), a measurable indicator of epithelial integrity. When purified VacA (acid-activated) was incubated with polarized host cell monolayers, it was shown to decrease TER significantly [89] and may contribute to epithelial barrier disruption induced by *Hp* (Figure 3E). This effect was confirmed in several types of polarized epithelial cells including EPH4, T84 and MDCK and during infection. The latter effect was independent of VacA’s vacuolating activity as it was also seen with m2-alleles of VacA [90]. It was proposed that selective permeabilization of polarized epithelial monolayers by VacA may lead to the release of certain molecules such as Ni^2+^, Fe^3+^, sugars and amino acids, which might support the growth of *Hp* withinthe gastric mucus [89,90]. As yet, the mechanism by which VacA alters paracellular permeability is little understood. However, it has also been noted that VacA increases the transepithelial flux of certain molecules such as urea the substrate of *Hp* urease [91]. The release of urea from cells, however, is attributed to the formation of VacA channels in the plasma membrane. Other studies have indicated that disruption of cell-to-cell junctions in polarized MDCK cells required VacA but also a second factor, injected CagA (Figure 3E), which targets both tight and adherens junctions [20,92]. In contrast, it was shown that incubation of polarized T84 epithelia by VacA prepared from broth culture filtrate of *Hp* did not modify the TER of the epithelia [93]. More complexity arises from another recent study showing that *Hp* induced a progressive loss of barrier function in INS-GAS mice and MKN-28 gastric epithelial cells, which was attenuated by inactivation of the Δ*ureB* gene, but not Δ*vacA* or ΔT4SS genes [94]. Remarkably, *Hp* induced the deregulation of specific tight junction proteins, including phosphorylation of MLC (myosin regulatory light chain) by MLC kinase and internalization of occludin. Thus, epithelial barrier disruption by *Hp* is a highly complex scenario, requiring various bacterial factors and signalling pathways which still need to be investigated in more detailed future studies [95].

## 8. VacA Elicits Suppressive Activities on Immune Cell Proliferation

During the long co-evolution of *Hp* with humans, the bacteria have accumulated numerous strategies to circumvent host immune cell functions. Some of these functions have been attributed to VacA (Figure 3J-L). A pioneering early study demonstrated that VacA can inhibit processing and presentation of antigenic peptides to human CD4-positive T cells (Figure 3J), and suggested for the first time that VacA may contribute to the persistence of *Hp* by interfering with protective immune responses [96]. In infected professional phagocytes, such as mouse RAW 264.7 and human THP-1 macrophage-like cell lines, wild-type *Hp* exhibited enhanced survival as compared to *vacA* deficient mutants [97]. Therefore, it appears that VacA can arrest phagosome maturation by recruiting and retaining coronin-1 or TACO, the tryptophane aspartate-containing coat protein (Figure 3K). In addition, it was shown that VacA can inhibit the expression of endothelial nitric oxygen synthase (eNOS) which resulted in significantly less production of reactive oxygen species (ROS) in monocyte/macrophage-like U937 cells and enhanced survival of VacA-positive *Hp* (Figure 3J). The detected pathway involved a suppressive effect of VacA on the expression of ILK (integrin-linked kinase), which provided a direct link to eNOS downsteam of ILK [98]. However, enhanced survival of isogenic Δ*vacA* mutants as compared to their wild-type strains was not seen in two other reports when freshly isolated human monocytes were infected [99,100]. The latter differences may be attributed to varying infection doses, cell types or *Hp* strains carrying different *vacA* alleles.

Co-incubation of purified VacA with or infection of T lymphocytes yields multiple effects. For example, VacA can specifically block the antigen-dependent proliferation of Jurkat T cells by interfering with IL-2-mediated signalling [101,102]. As discussed above, *Hp* targets CD18 (β2 integrin) as the receptor of VacA on human T lymphocytes [62]. After entry, VacA inhibits Ca^2+^ mobilization and, subsequently, downregulation of the activity of the Ca^2+^-dependent phosphatase calcineurin. This in turn inhibits the activation of transcription factor NFAT (nuclear factor of activated T cells). As a consequence, NFAT target genes such as IL-2 and the high-affinity IL-2 receptor (IL-2R-alpha) are not expressed anymore (Figure 3L). VacA, however, exerts a different effect on primary human CD4-positive T cells, whose proliferation is inhibited through the T cell receptor (CD28) and eventually by other VacA activities [103]. In the latter study, VacA was shown to suppress IL-2-induced cell cycle progression and proliferation of primary T cells without affecting NFAT. Therefore, VacA may inhibit the clonal expansion of T cells that have been activated by numerous bacterial antigens. This in turn would allow *Hp* to evade adaptive immune responses which can permit persistent infection. 

## 9. VacA-Dependent Pro-Inflammatory Signalling Activities

Numerous *Hp* factors have been implicated in the induction of gastric inflammation. Besides peptidoglycan [14], OipA [13] and CagA [21], VacA has now also been found to activate pro-inflammatory responses during infection with *Hp*. For example, addition of VacA to the gastric adenocarcinoma cell line AZ-521 activates two classes of MAP kinases (p38 and Erk-1/2) and the activating transcription factor 2 (ATF-2) signalling pathway [104], (Figure 3B). Pharmacological inhibition of p38 kinase activity, however, did not block the vacuolation phenotype or mitochondrial damage, which indicates that VacA-induced activation of the p38/ATF-2 signalling cascade is independent of endosomal and mitochondrial pathways of VacA [104]. VacA has also been reported to induce activation of p38 in several other types of cells [102] and U937 macrophage-like cells [105]. In the latter study, VacA-induced p38 activation in U937 cells revealed a prominent role in stimulation IL-8 production via ATF-2, cAMP response element binding (CREB) protein and nuclear transcription factor kappa B (NF-κB). Interestingly, VacA can also induce NF-κB in T cells [106], thus the toxin obviously has two paradoxical activities on T cells, immunosuppression and pro-inflammatory effects (Figure 3J). In addition, in neutrophils and macrophages, VacA treatment can stimulate the expression of Cox-2 (cyclooxygenase-2), a pro-inflammatory enzyme [102]. The expression of Cox-2 is mediated by VacA-induction of p38 and also enhanced the secretion of prostaglandin E2 [105]. In another cell culture model, binding of VacA to RBL-2H3 mast cells resulted in a rapid change in cytosolic Ca^2+^ concentrations [107]. This was accompanied by stimulation of pro-inflammatory cytokines (including IL-6 and TNF-α), resulting in chemotaxis and degranulation of these mast cells [107,108]. 

## 10. Molecular Crosstalk between VacA and CagA Signalling Pathways

Several recent studies have demonstrated that VacA does not act independently of other bacterial colonization or virulence factors. There are four prime examples of molecular crosstalk between VacA, CagA and the T4SS affecting multiple responses including *Hp*-induced apoptosis, cell vacuolation, cell elongation, NFAT signalling and EGFR activation, which will be discussed below.

### 10.1. Role of VacA and CagA in Apoptotic and Anti-Apoptotic Signalling

As described above VacA can target mitochondria and induce apoptotic cell death *in vitro*. However, for gastric cell apoptosis *in vivo*, several other parameters are likely to be important. Under normal conditions, rapid self-renewal of the gut epithelium, which occurs by a balance of progenitor proliferation and pit cell apoptosis, serves as a host defence mechanism to limit bacterial colonization. In the Mongolian gerbil (Meriones *unguiculatus*) model system, it was shown that *Hp* inhibits the apoptotic loss of pit cells [22]. Suppression of apoptosis contributed to pit hyperplasia and persistent bacterial colonization of the stomach, and this was mediated by injected CagA which stimulated the pro-survival mitogen-activated protein (MAP) kinase member Erk-1/2 and anti-apoptotic protein MCL-1 in the gastric pits. Thus, it appears that CagA counteracts VacA signalling and activates anti-apoptotic and host cell survival signal cascades to overcome self-renewal of the gastric epithelium and contribute to chronic *Hp* infection [22]. In a very recent study, the transfected carboxy-terminal domain of CagA (38 kDa) inhibited VacA-induced apoptosis by two complementary mechanisms [109]. Tyrosine-phosphorylated CagA prevented pinocytosed VacA to reach its target intracellular compartments. Unphosphorylated CagA triggered an anti-apoptotic activity blocking VacA-induced apoptosis at the mitochondrial level without affecting the intracellular trafficking of the toxin. These results were confirmed during infection with *Hp* and it was proposed that, once bacteria have colonized the gastric niche, the apoptotic action of VacA might be detrimental for the survival of *Hp* adherent to the mucosa. Thus, CagA association with VacA maybe a novel, highly ingenious microbial strategy to locally protect its ecological niche against a bacterial virulence factor, with however detrimental consequences for the human host [109]. 

### 10.2. Role of VacA and CagA in NFAT Signalling

It has been demonstrated that CagA and VacA have opposing effects in T cells, for example on the activation of transcription factor NFAT [110]. A genome-wide screening of CagA-responsive genes using DNA microarrays identified NFAT transcription factors whose binding sites were overrepresented in the promoter regions of CagA-activated genes. The results of reporter assays confirmed that CagA was capable of activating NFAT in a manner independent of CagA phosphorylation. Expression of CagA in gastric epithelial cells provoked translocation of NFATc3, a member of the NFAT family, from the cytoplasm to the nucleus and activated an NFAT-regulated gene, p21WAF1/Cip1. CagA-mediated NFAT activation was abolished by inhibiting calcineurin or phospholipase C-gamma activity. Furthermore, treatment of gastric epithelial cells with purified VacA, which inhibits NFAT activity in T lymphocytes [101], counteracted the ability of CagA to activate NFAT [110]. These findings indicate that the two major *Hp* virulence factors inversely control NFAT activity. Considering the pleiotropic roles of NFAT in cell growth and differentiation, deregulation of NFAT, either positively or negatively, depending on the relative exposure of cells to CagA and VacA, may contribute to the various disease outcomes caused by *Hp* infection. 

### 10.3. Role of VacA and CagA in the Vacuolation and Cell Elongation Phenotypes

It has been demonstrated *in vitro* that VacA can negatively regulate the CagA-induced elongation phenotype, and CagA can downregulate VacA-induced cell vacuolation [111,112]. In particular, isogenic Δ*cagA* and T4SS-deficient *Hp* mutants were found to significantly increase VacA-induced vacuolation of AGS gastric epithelial cells, and the Δ*vacA* mutants significantly increased the CagA-induced cellular elongation phenotype as compared with their wild-type strains. Although AGS cells incubated with the wild-type *Hp* strains may display both vacuolation and elongation phenotype formation, it was found that (i) the length of elongated cells was significantly reduced in vacuolated cells compared with those without vacuolation; (ii) the number of vacuoles was significantly reduced in elongated cells; and (iii) cells displaying extensive vacuolation did not subsequently elongate and vice versa. Importantly, VacA did not affect the phosphorylation of CagA [111,112]. These data indicate that VacA and CagA can downregulate each other's phenotypical effects on AGS epithelial cells.

### 10.4. Role of VacA and T4SS in Epidermal Growth Factor Receptor (EGFR) Signalling

Another recent study indicated that the T4SS and VacA have opposing effects on the activation of members of the EGFR family kinases during infection with *Hp* [112]. Early studies have demonstrated that *Hp* can activate EGFR in a T4SS-dependent manner [24,25], and that addition of purified VacA or VacA-positive culture supernatants had suppressive effects on proliferation, migration or wound healing of gastric epithelial cells *in vitro* [113,114,115,116]. In the latter studies it was shown that VacA can block the activity of externally added EGF. These findings suggest that VacA may interfere with EGFR receptor signalling and cell proliferation (Figure 3G,H). Indeed, preparations of VacA or infection with VacA-expressing *Hp* exhibited suppressive effects on the phosphorylation and consequently activation of EGFR [112,114] and HER2/Neu, another member of the EGFR family [112]. Immunofluorescence data showed internalized EGFR in infections with VacA-expressing *Hp*,providing a possible mechanism for how significant portions of EGFR are inactivated by VacA [112]. Interestingly, RPTP-alpha but not vacuolation *per se* played a role in this process. In addition, it was shown that EGFR-mediated Erk-1/2 activation is necessary for the CagA-induced elongation phenotype (Figure 3I), which can be suppressed in infection with *Hp* strains expressing highly active VacA [112]. Although further investigation is necessary, these data clearly demonstrate that VacA can downregulate CagA's effects on epithelial cells, a novel molecular mechanism showing how *Hp* may avoid excessive cellular damage during infection, which may also contribute to ensure persistent infection. 

## 11. Other VacA-Dependent Signalling Activities

In addition to the VacA-triggered activities described above, several other features have been described. For example, Yeast two-hybrid (Y2H) screens and other experiments have demonstrated VacA’s potential for specific binding to the receptor for activated C kinase-1, Rack-1 (Table 1), however, its function for the infection process is widely unknown [69]. The effect of the toxin on endosomal and lysosomal functions was also studied by following procathepsin-D maturation and EGF degradation in HeLa cells exposed to VacA. Interestingly, VacA blocked cleavage of procathepsin-D (53 kDa) into both the intermediate (47 kDa) and the mature (31 kDa) form, and intracellular degradation of EGF was also inhibited [117]. These findings suggest that VacA can also impair the degradative power of late endosomes and lysosomes. Finally, further complexity in the overall scenario arises from the observation that VacA can also activate the beta-catenin signalling pathway [118]. Incubation of AZ-521 cells with VacA resulted in phosphorylation of protein kinase B (Akt) and glycogen synthase kinase-3beta (GSK3beta) through a phosphoinositide-3-kinase (PI3K)-dependent pathway. Following phosphorylation and inhibition of GSK3beta, beta-catenin was released from a GSK3beta/beta-catenin complex, with subsequent nuclear translocation. Inhibitor studies indicated that VacA-induced phosphorylation of Akt does not require VacA internalization and is independent of vacuolation. In contrast, infection with a Δ*vacA* mutant failed to induce phosphorylation of Akt and GSK3beta, or release of beta-catenin from a GSK3beta/beta-catenin complex [118]. These results support the hypothesis that VacA activates the PI3K→Akt signalling pathway, resulting in phosphorylation and inhibition of GSK3beta. Subsequent translocation of beta-catenin into the host cell nucleus is consistent with effects of VacA on beta-catenin-regulated transcriptional activity and a new possible role in the pathogenesis of *Hp*, including the development of gastric cancer. Thus, VacA is a highly fascinating bacterial toxin with multiple signalling activities. 

## 12. Conclusions

*Hp* is the most common chronic bacterial infection worldwide, and, although asymptomatic in the majority of infected persons, it is also the cause of significant rates of morbidity and mortality. The interest in this field is underlined by more than 28,000 scientific publications that have appeared in PubMed since its discovery in 1982. However, *Hp* infection occurs in approximately half of the world population, with disease being an exception rather than the rule. Understanding how this organism interacts with its host is essential for formulating an effective strategy to deal with its most important clinical consequences. Studies of host-bacterial interactions and bacterial virulence factors in this infection model have provided important insights for clinical practice. Research over the past decade on VacA, CagA and the T4SS has provided us with fundamental insights into *Hp* biology and has generated a complex and often puzzling scenario. VacA is secreted into the extracellular space and also is, at least in part, retained on the bacterial cell surface. The toxin exists in monomeric and oligomeric forms, and binds to multiple host cell surface receptors. We have discussed here the cellular effects induced by VacA which include pore formation in the plasma membrane, alteration of endo-lysosomal function, vacuolation, apoptosis and immune cell inhibition. VacA has been demonstrated to target several different cell components, including endocytic vesicles, the actin-cytoskeleton and mitochondria. However, it still remains unclear whether VacA has an enzymatic activity, and if it should be categorised as an A/B-type toxin, a pore-forming toxin or both. In addition, it appears that there is substantial molecular crosstalk between VacA and CagA signalling cascades during infection both in epithelial and immune cells. These novel findings further highlight the enormous complexity in *Hp*-host cell interactions. Thus, it appears that *Hp*‘s virulence factors will continue to be a fascinating and rewarding research subject in future studies.
